# Activation of the Nrf2-Keap 1 Pathway in Short-Term Iodide Excess in Thyroid in Rats

**DOI:** 10.1155/2017/4383652

**Published:** 2017-01-04

**Authors:** Tingting Wang, Xue Liang, Iruni Roshanie Abeysekera, Umar Iqbal, Qi Duan, Gargi Naha, Laixiang Lin, Xiaomei Yao

**Affiliations:** ^1^Department of Physiology and Pathophysiology, School of Basic Medical Sciences, Tianjin Medical University, Tianjin 300070, China; ^2^Key Laboratory of Hormones and Development (Ministry of Health), Tianjin Key Laboratory of Metabolic Diseases, Tianjin Metabolic Diseases Hospital & Tianjin Institute of Endocrinology, Tianjin Medical University, Tianjin 300070, China

## Abstract

Wistar rats were randomly divided into groups of varying iodide intake: normal iodide; 10 times high iodide; and 100 times high iodide on Days 7, 14, and 28. Insignificant changes were observed in thyroid hormone levels (*p* > 0.05). Urinary iodine concentration and iodine content in the thyroid glands increased after high consumption of iodide from NI to 100 HI (*p* < 0.05). The urinary iodine concentration of the 100 HI group on Days 7, 14, and 28 was 60–80 times that of the NI group. The mitochondrial superoxide production and expressions of Nrf2, Srx, and Prx 3 all significantly increased, while Keap 1 significantly decreased in the 100 HI group when compared to the NI or 10 HI group on Days 7, 14, and 28 (*p* < 0.05). Immunofluorescence staining results showed that Nrf2 was localized in the cytoplasm in NI group. Although Nrf2 was detected in both cytoplasm and nucleus in 10 HI and 100 HI groups, a stronger positive staining was found in the nucleus. We conclude that the activation of the Nrf2-Keap 1 antioxidative defense mechanism may play a crucial role in protecting thyroid function from short-term iodide excess in rats.

## 1. Introduction

Iodine being a critical constituent of thyroid hormones is essential for normal growth and development in all vertebrates [1, 2]. During thyroid hormone synthesis, there is a constant production of reactive oxygen species (ROS), especially hydrogen peroxide (H_2_O_2_), which is subsequently utilized for the oxidation of iodide [3]. Although the basal level of ROS production is important for maintaining thyroid hormone biosynthesis, iodide excess may increase the production of ROS in thyrocytes [4, 5]. Higher amounts of ROS can cause oxidative stress by damaging the cellular components and affecting organelle integrity [5]. The increased generation of ROS triggered by iodide excess is responsible for its cytotoxic effect on thyrocytes [6, 7].

Thomopoulos reported that hyperthyroidism may develop in around 10% of patients with excess iodine and that it may occur several years after the initiation of iodine excess [8]. Wolff reported that chronic ingestion of more than ten times the daily requirement of iodide or iodide-generating organic compounds could lead to iodide goiter in certain subjects [9]. In the thyroid slices of several species, excess iodide is known to stimulate the generation of H_2_O_2_ [10]. This occurs when the latter is in the presence of either 300 *μ*M KI in dog thyroid slices or 100 *μ*M KI in bovine thyroid slices [10].

Possible molecular mechanisms responsible for excess iodide-induced ROS production are described as below. When iodide is in excess as compared with tyrosine residues, it reacts with the iodonium cation formed by iodide oxidation to give molecular iodine. Excess molecular iodine induces apoptosis through an increased generation of free radicals [11]. Various types of iodolipids are produced when iodine binds to membrane lipids, which is considered to be the main mechanism of free radical-induced damage. The mitochondria contain specific receptors for the thyroid hormones, and much of the ROS production occurs here via oxidative phosphorylation [3, 12]. ROS include free radicals, such as superoxide anions, hydroxyl radicals, and H_2_O_2_.

Nuclear factor erythroid 2-related factor 2 (Nrf2) is a transcription factor, which is vital in regulating the expression of some antioxidative enzymes, such as hemeoxygenase-1, thioredoxin, peroxiredoxins (Prxs), and Sulfiredoxin (Srx) [13]. Nrf2 is released from the Nrf2-Keap 1 (Kelch-like ECH-associated protein 1) complex and translocated to the nucleus after the initiation of oxidative stress [14]. Srx, a recently discovered member of the oxidoreductases family, contributes to cellular redox balance. Previous studies have shown Srx to be the only enzyme which catalyses ATP-dependent reduction of the hyperoxidized form of Prxs [15, 16]. Prxs are important peroxidases that reduce peroxides [17, 18]. Peroxiredoxin 3 (Prx 3) is a critical scavenger for mitochondrial H_2_O_2_. Also, mitochondria contain 30 times more Prx 3 than glutathione peroxidase [19].

In the present study, we aim to investigate the effect of normal iodide intake (NI), 10 times high iodide intake (10 HI), and 100 times high iodide intake (100 HI) on Days 7, 14, and 28 on the antioxidative action of Srx and Prx 3 via Nrf2-Keap 1 pathway in the thyroid of rats.

## 2. Methods

### 2.1. Animals and Diet

A total of 216 Wistar rats (eight weeks old) at SPF level, weighing 296.36 ± 8.53 g, were randomly assigned to NI, 10 HI, and 100 HI groups. Along with the normal diet, the NI group (with the addition of deionized water), 10 HI and 100 HI groups received different dosages of potassium iodide in the deionized water, resulting in the following daily iodide intake: 7.5 *μ*g/d, 75 *μ*g/d, and 750 *μ*g/d, respectively [20]. The rats were sacrificed after iodide intake for a week, two weeks, and four weeks at ages of 9, 10, and 12 weeks, respectively (they are collectively referred to as Day 7, Day 14, and Day 28). In this study, a total of two rats died in the NI group, and none died in any other group. Animal procedures were approved by the Institutional Animal Care and Use Committee of Tianjin Medical University (the number is SYXK (Jin): 2014-0004), which is in accordance with the NIH Guide.

### 2.2. Reagent

Anti-Peroxiredoxin-3 antibody (ab16751) and anti-Keap 1 antibody (ab66620) were purchased from Abcam (Abcam, Cambridge, MA, USA). Nrf2 (H-300): sc-13032, Sulfiredoxin (FL-137): sc-99076, goat anti-rabbit IgG-HRP: sc-2004, goat anti-mouse IgG-HRP: sc-2005, and goat anti-rabbit IgG-PE: sc-3739 were bought from Santa Cruz (Santa Cruz Biotechnology, Inc., CA, USA). MitoSOX Red (3,8-phenanthridinediamine,5-(6′-triphenylphosphoniumhexyl)-5,6-dihydro-6-phenyl) mitochondrial superoxide indicator (M36008) was purchased from Invitrogen (Invitrogen Life Technologies, CA, USA). *β*-Actin (AA128) was purchased from Beyotime (Beyotime Institute of Biotechnology, Jiangsu, China). RPMI-1640 and fetal bovine serum (FBS) were purchased from GE Healthcare Life Sciences (HyClone, UT, USA). Immobilon Western Chemiluminescent HRP Substrate (WBKLS0100) was purchased from Millipore (Merck Millipore, MA, USA). All the other chemicals made in China were of analytic grade [21].

### 2.3. Thyroid Weight Measurement

Following the intake of NI, 10 HI, and 100 HI, the body weight and the thyroid weight were measured when the rats were sacrificed on Days 7, 14, and 28. We calculated the ratio of the thyroid weight/body weight (milligrams per 100 gram body) according to the ratio of the viscera (viscera/body weight) [21, 22]. The rats were anesthetized at appropriate concentrations (10% chloral hydrate, 0.3 mL/100 g). Skin, subcutaneous tissue, fascia, and muscles of the anterior neck were removed and the thyroid gland was exposed. In order to ensure the integrity of the thyroid gland, it was removed carefully with a trachea ring. The fascia covering thyroid gland was stripped and the thyroid gland was collected under stereoscopic microscope carefully.

### 2.4. Measurement of Urinary Iodine Concentration and Iodine Content in the Thyroid Glands

Urine samples were collected using metabolic cages for 24 hours the day before the rats were sacrificed. Thyroid tissue homogenates were prepared. Urinary iodine concentration and iodine content in the thyroid glands were measured by As-Ce catalytic spectrophotometry in the Key Lab of Hormones and Development Ministry of Health, Institute of Endocrinology, Tianjin Medical University [23]. The iodine standard solution and samples were added to the test tubes (15 mm × 150 mm), respectively. 1 mL ammonium persulfate was added and then mixed and digested for 60 minutes at 100°C. After the test tubes were cooled down, 2.5 mL of arsenious acid solution was added and mixed. Consequently, 0.3 mL cerium sulfate solution was added and mixed every 30 s. The absorbance at 400 nm was measured with a spectrophotometer.

### 2.5. Serum Thyroid Hormones Levels Measurement

Blood samples were taken from the carotid artery and then centrifuged for 10 min at 2000 r/min to obtain the samples of the serum. After that, the samples were stored at −80°C for further analysis. Levels of serum total thyroxine (TT4), total triiodothyronine (TT3), free thyroxine (FT4), and free triiodothyronine (FT3) were all determined using a chemiluminescent immunoassay technique. All kits for thyroid function were purchased from Siemens (Siemens Healthcare Diagnostics Products Limited, Llanberis, UK).

### 2.6. Flow Cytometry

MitoSOX Red was used to measure mitochondrial superoxide production by flow cytometry. The thyroid cell suspension of Wistar rats was prepared. 5 *μ*M of MitoSOX Red was added and the suspension was incubated for 10 min at 37°C in the dark. Flow cytometry was carried out using a FACSCalibur (BD Bioscience, San Jose, CA). Collecting FL2 channel forward scattering (forward scatter, FSC) and lateral scattering (side scatter, SSC) data, 10000 cells were collected for each sample. The control group without MitoSOX was regarded as the blank zero group for standardization [24].

### 2.7. Western Blot Analysis

The bicinchoninic acid protein assay kit (Beyotime Institute of Biotechnology, Jiangsu, China) was used to detect the concentration of proteins. 50 *μ*g proteins were separated by SDS-PAGE and transferred to the PVDF membrane. Subsequently, the membrane was blocked for 1 hour at room temperature using 5% nonfat milk. Then the membrane was incubated overnight at 4°C with primary antibodies followed by horseradish peroxidase conjugated secondary antibodies. The proteins were detected by Immobilon Western Chemiluminescent HRP Substrate. *β*-Actin was used as a loading control. Blots were scanned as gray scale images and quantified using Image J software (NIH). All the blot intensities were normalized with that of the loading control *β*-actin.

### 2.8. Immunofluorescence Staining

The thyroid tissues were first embedded with an Optimal Cutting Temperature (OCT) compound at −80°C. Following which the tissues were frozen in a cryostat machine and cut into frozen sections for 5 *μ*m at −20°C. The slices were incubated with 5% FBS for 60 min at room temperature. Subsequently, they were incubated with a primary antibody [Nrf2 (1 : 100) or Srx (1 : 100)] at 4°C, overnight. After three washes with PBS, the second antibody was linked to fluorophores (goat anti-rabbit IgG-PE). The nucleus was stained for 5 min with Hoechst 33258 (50 *μ*L) and washed 3 times with PBS. MitoSOX Red was used to measure mitochondrial superoxide production. Using a Zeiss LSM 510 confocal microscope, fluorescent images of the prepared slides were obtained.

### 2.9. Statistics

The data of urinary iodine concentration (Table 1) showed a skewed distribution and were expressed as the median. Differences between groups were evaluated by nonparametric Kruskal–Wallis test. If the latter test showed significant differences between groups, the individual groups were compared with the control group by the Nemenyi tests using SPSS 22.0. A *p* value of <0.05 was considered statistically significant [25].

The other data was expressed as mean ± SD. Differences between groups were evaluated by one-way analysis of variance (ANOVA); if this test showed significant differences between groups, the individual groups were compared with the control group by Least Significant Difference (LSD) test using SPSS 22.0. A *p* value of <0.05 was considered statistically significant.

## 3. Results

### 3.1. Effects of the Iodide Intake (NI, 10 HI, and 100 HI) on the Ratio of Thyroid Weight/Body Weight on Days 7, 14, and 28

**Figure 1:**
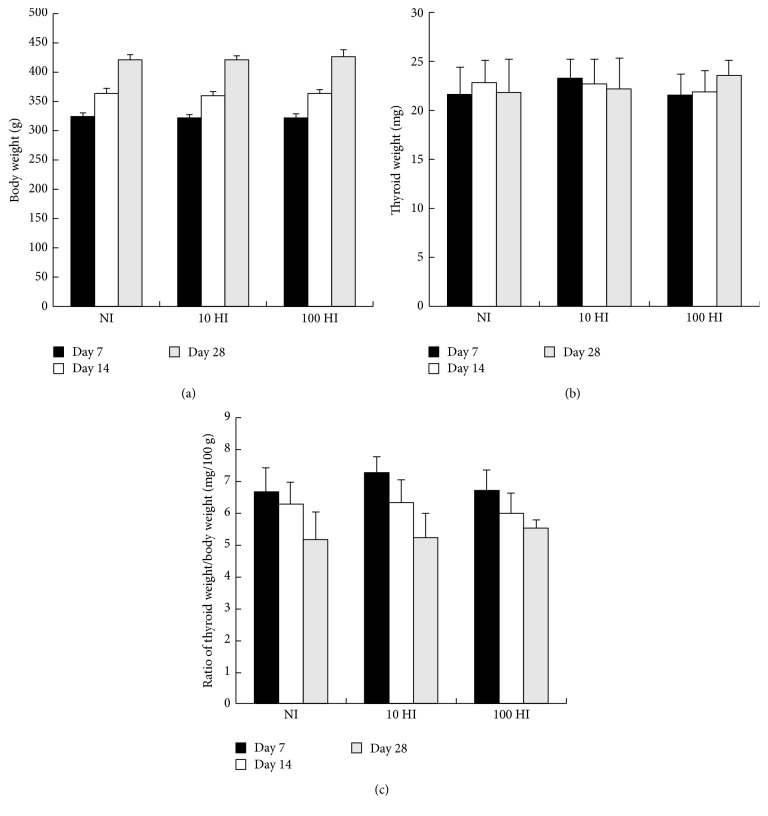
Effects of the iodide intake (NI, 10 HI, and 100 HI) on (a) the body weight; (b) the thyroid weight; and (c) the ratio of the thyroid weight/body weight on Days 7, 14, and 28. All data is presented as mean ± SD (*N* = 6 for each group). Statistical analyses were performed by one-way analysis of variance (ANOVA) with the Least Significant Difference (LSD) test.

The parameters body weight, thyroid weight, and the ratio of the thyroid weight/body weight were not significantly altered following the intake of NI, 10 HI, and 100 HI on Days 7, 14, and 28 (*p* > 0.05) (Figure 1).

### 3.2. Effects of the Iodide Intake (NI, 10 HI, and 100 HI) on Urinary Iodine Concentration and Iodine Content in the Thyroid Glands on Days 7, 14, and 28

The median urinary iodine concentration and their ranges for NI, 10 HI, and 100 HI on Days 7, 14, and 28 are illustrated in Table 1. In all the different time periods, there was a significant increase in the urinary iodine concentration among 10 HI and 100 HI when compared to the NI group (*p* < 0.05). In addition, the urinary iodine concentration of the 100 HI group also increased significantly when compared to 10 HI (*p* < 0.05). Between any of the iodide intake groups (NI, 10 HI, and 100 HI), there was no significant difference in the urinary iodine concentration on Day 14 and Day 28 when compared to Day 7 (*p* > 0.05). Furthermore, there was no significant difference between Day 14 and Day 28 (*p* > 0.05). When the intake of iodide was increased from NI to 10 HI and further to 100 HI, a simultaneous increase in the urinary iodine concentration was also observed on Days 7, 14, and 28. The urinary iodine concentration in the 10 HI group was approximately 10 times that of the NI group, whereas the urinary iodine concentration in the 100 HI group was about 60–80 times that of the NI group on Days 7, 14, and 28.

In our study, we demonstrated that the iodine content in the thyroid glands was significantly increased in the 100 HI group when compared to the NI group on Days 7, 14, and 28 (*p* < 0.05). Between any of the iodide intake groups (NI, 10 HI, and 100 HI), there was no significant difference found in the iodine content in the thyroid glands on Days 7, 14, and 28 (*p* > 0.05). When the intake of iodide was increased from NI to 10 HI and further to 100 HI, the iodine content in the thyroid glands increased gradually on Days 7, 14, and 28 (Table 2).

### 3.3. Effects of the Iodide Intake (NI, 10 HI, and 100 HI) on the Changes of Serum Thyroid Hormones Levels on Days 7, 14, and 28

There were no significant alterations in TT3, TT4, FT3, and FT4 levels following the intake of NI, 10 HI, and 100 HI on Days 7, 14, and 28 (*p* > 0.05). Moreover, for all three dosages of iodide intake, there were no significant differences in any of the serum thyroid hormones levels on Day 14 and Day 28 when compared to Day 7 (*p* > 0.05). In addition, there were no significant changes between Day 14 and Day 28 in serum thyroid hormones levels (TT3, TT4, FT3, and FT4) (*p* > 0.05) (Table 3).

### 3.4. Effects of the Iodide Intake (NI, 10 HI, and 100 HI) on the Changes of Mitochondrial Superoxide Production on Days 7, 14, and 28

**Figure 2:**
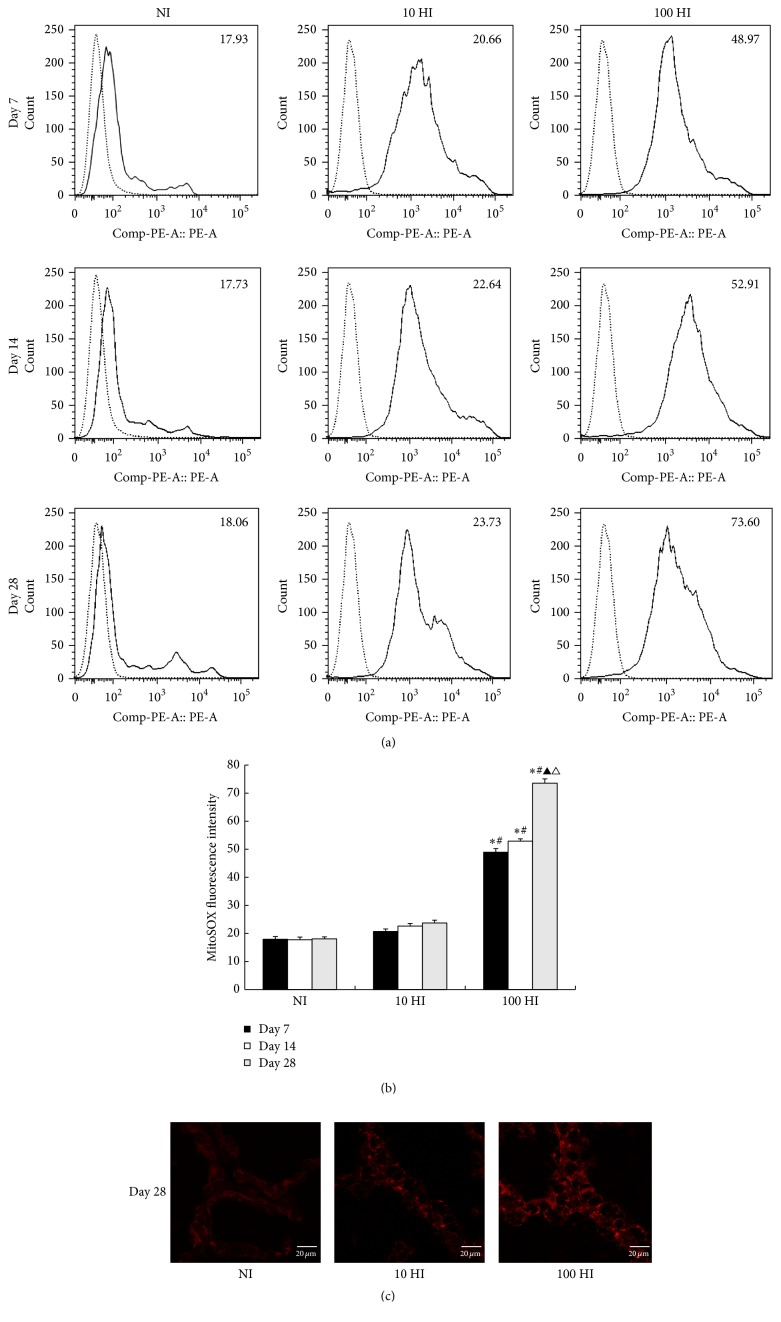
Effects of the iodide intake (NI, 10 HI, and 100 HI) on the changes of mitochondrial superoxide production on Days 7, 14, and 28. (a) There was a significant increase in the mitochondrial superoxide production in the 100 HI group on Days 7, 14, and 28 (*p* < 0.05). The difference was more significant on Day 28 when compared to Day 7 or Day 14. (b) Histogram analysis was performed on the mean fluorescence intensity of MitoSOX Red. All data is presented as the mean ± SD (*N* = 6 for each group). Statistical analyses were performed by one-way analysis of variance (ANOVA) with the Least Significant Difference (LSD) test. ^*∗*^ *p* < 0.05 versus the NI group on Days 7, 14, and 28, respectively. ^#^ *p* < 0.05 versus the 10 HI group on Days 7, 14, and 28, respectively. ^▲^ *p* < 0.05 versus the Day 7 group in the 100 HI group. ^△^ *p* < 0.05 versus the Day 14 in the 100 HI group. Experiments were repeated 3 times with similar results. (c) There was a significant increase in the mitochondrial superoxide production in the 100 HI group on Day 28 and was observed by confocal microscopy. Scale bar: 20 *µ*m.

On Days 7, 14, and 28, when compared to the NI group, the mitochondrial superoxide production in the 10 HI group showed no significant increase (*p* > 0.05). However, there was a significant increase in the mitochondrial superoxide production in the 100 HI group (*p* < 0.05). When compared to the 10 HI, there was a significant increase in the mitochondrial superoxide production in the 100 HI group (*p* < 0.05). In the NI and 10 HI groups, the mitochondrial superoxide production on Days 7, 14, and 28 showed no significant difference (*p* > 0.05). However, in the 100 HI group, compared to Day 7, there was a significant increase in mitochondrial superoxide production on Day 28 (*p* < 0.05). Similarly, compared to Day 14, there was also a significant increase in mitochondrial superoxide production on Day 28 (*p* < 0.05). Accordingly, the fluorescent intensity of MitoSOX Red on Day 28 gradually increased after the increased dosages of iodide intake from NI to 100 HI, which was consistent with our results of flow cytometry (Figure 2).

### 3.5. Effects of the Iodide Intake (NI, 10 HI, and 100 HI) on the Changes of Nrf2, Keap 1, Srx, and Prx 3 Expressions on Days 7, 14, and 28

**Figure 3:**
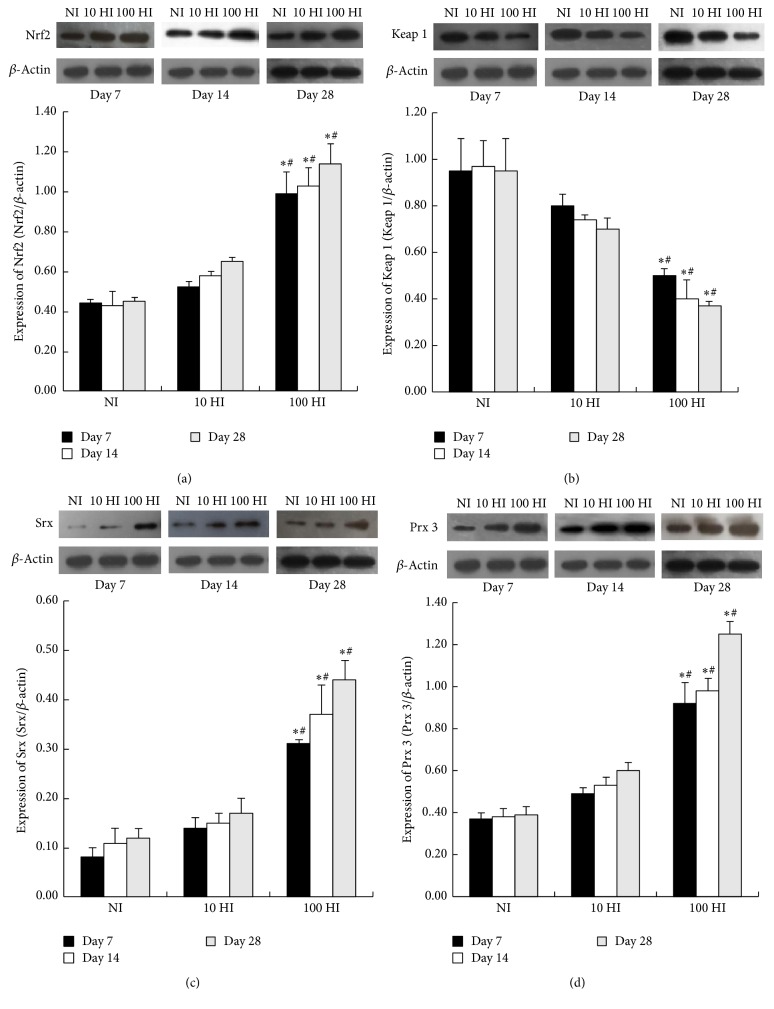
Effects of the iodide intake (NI, 10 HI, and 100 HI) on the changes of (a) Nrf2; (b) Keap 1; (c) Srx; and (d) Prx 3 expressions on Days 7, 14, and 28. Representative western blot and histograms of densitometric analyses normalized for the relative *β*-actin content. All data is presented as the mean ± SD (*N* = 6 for each group). Statistical analyses were performed by one-way analysis of variance (ANOVA) with the Least Significant Difference (LSD) test. ^*∗*^ *p* < 0.05 versus the NI group on Days 7, 14, and 28, respectively. ^#^ *p* < 0.05 versus the 10 HI group on Days 7, 14, and 28, respectively. Experiments were repeated 3 times with similar results.

On Days 7, 14, and 28, when compared to the NI group, the expressions of Nrf2, Keap 1, Srx and Prx 3 showed no significant differences in the 10 HI group (*p* > 0.05); the expressions of Nrf2, Srx, and Prx 3 were significantly increased while Keap 1 was notably decreased in the 100 HI group (*p* < 0.05). On Days 7, 14, and 28, when compared to the 10 HI group, the expressions of Nrf2, Srx, and Prx 3 were significantly increased while Keap 1 was significantly decreased in the 100 HI group (*p* < 0.05) (Figure 3).

### 3.6. Effects of the Iodide Intake (NI, 10 HI, and 100 HI) on the Changes of Immunofluorescence Staining on Days 7, 14, and 28

**Figure 4:**
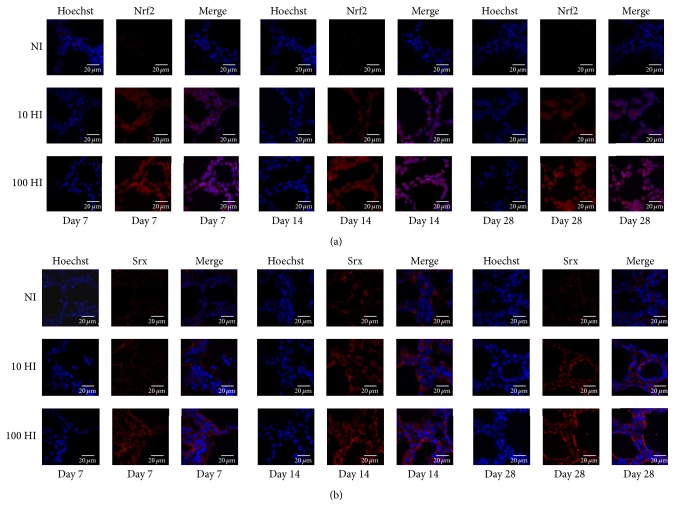
Effect of the iodide intake (NI, 10 HI, and 100 HI) on the changes of immunofluorescence staining on Days 7, 14, and 28. (a) In the NI group, Nrf2 (red) was localized in the cytoplasm on Days 7, 14, and 28. In the 10 HI group, the positive staining of Nrf2 can be observed in both the nucleus and the cytoplasm. Moreover, in the 100 HI group, a stronger positive staining of Nrf2 can be detected in the nucleus on Days 7, 14, and 28; the nucleus was dyed with Hoechst (blue). (b) Srx (red) positive staining was located in the cytoplasm; the nucleus was dyed with Hoechst (blue). Scale bar: 20 *μ*m.

Following the increased iodide intake from NI to 10 HI and further to 100 HI, the expressions of Nrf2 and Srx intensified. In the NI group, Nrf2 was localized in the cytoplasm on Days 7, 14, and 28. In the 10 HI group, the positive staining of Nrf2 can be observed in both the nucleus and the cytoplasm. Moreover, in the 100 HI group, a stronger positive staining of Nrf2 can be detected in the nucleus on Days 7, 14, and 28 (Figure 4(a)). Srx positive staining was only located in the cytoplasm on Days 7, 14, and 28 (Figure 4(b)).

## 4. Discussion

In our study, we found that there were no significant alterations in TT3, TT4, FT3, and FT4 levels following the intake of NI, 10 HI, and 100 HI on Days 7, 14, and 28 (*p* > 0.05). There are very efficient homeostatic mechanisms that resist changes in circulating T3 and T4 levels in response to iodide excess. Due to the compensatory mechanisms, such as the Wolff-Chaikoff effect, the changes in T3 and T4 levels following an increase in iodide intake are minimal and usually transient in nature [26–28]. The Wolff-Chaikoff effect relies on a high (≥10^−3^ molar) intracellular concentration of iodide. During initial exposure, excess iodide is transported by the sodium-iodide symporter (NIS) into the cells. When intracellular concentration reaches at least 10^−3^ molar, iodide organification is blocked [27, 29]. Moreover, the regulatory mechanisms include modulation of blood flow, enzyme activity, gene expression, and transport proteins in signaling pathways [2, 30, 31]. In a study conducted by Eng et al., 16 Wistar rats were given 2000 *μ*g of iodide acutely; it was observed that there was a significant decrease in serum T4 and T3 levels on Day 1 of the study. Subsequently, on Day 6, both serum T4 and T3 levels returned to normal ranges. However, in both cases, the serum TSH levels remained unchanged [32]. Mooij et al. observed no significant changes in serum thyroid hormones levels when female Wistar rats were given 100 *μ*g iodide daily for 18 weeks [33]. Paul et al. demonstrated that when normal volunteers received 1500 *μ*g of supplemental iodine daily for 14 days, a small decrease in serum T3 and T4 concentrations with compensatory increase of TSH was detected, although all values remained within the normal ranges [34]. However, the presence of handicaps such as an increased autoimmune susceptibility, fetal period, extremes of age, pregnancy, lactation, or an active pathological entity significantly impair these mechanisms [1, 35–37].

Our study demonstrated that both the urinary iodine concentration and the iodide intake increased simultaneously from NI to 10 HI and further to 100 HI. Interestingly, we found that the urinary iodine concentration of the 10 HI group was approximately 10 times that of the NI group, whereas the urinary iodine concentration of the 100 HI group was about 60–80 times that of the NI group on Days 7, 14, and 28. The urinary iodine concentration is regarded as a sensitive indicator of iodine status because approximately 90% of ingested iodide is excreted in the urine [38, 39]. There is an increase for the maintenance of thyroid homeostasis as well as the steady state of the internal environment of the body. The thyroid gland has adaptation mechanisms that reduce iodide metabolism when the supply is abundant, thus avoiding thyrotoxicosis. There are several mechanisms which include a direct inhibitory effect of iodide in the thyroid itself and inhibition by iodide of its own organification (Wolff-Chaikoff effect), its transport, thyroid hormones secretion, cAMP formation in response to TSH, and several other metabolic steps [40]. We suggest that all the protective mechanisms may ensure the excessive iodide intake of the 10 HI group on Days 7, 14, and 28 be eliminated from urine. The urinary iodine concentration of the 100 HI group on Days 7, 14, and 28 was about 60–80 times that of the NI group, although the thyroid function was normal. This leads us to propose the idea that excessive iodide accumulated in the body may trigger the oxidative and antioxidative signaling pathway to maintain the normal thyroid function.

We demonstrated that the production of mitochondrial superoxide significantly increased on Days 7, 14, and 28 in the 100 HI group. This is consistent with our previous study on metallothionein-I/II knockout mice [24]. Joanta et al. reported that the initiation of free radical production was observed after giving a high dose of iodide [3]. Serrano-Nascimento et al. demonstrated that an increased mitochondrial superoxide production was shown in response to NaI (10^−6^ M to 10^−3^ M) treatment in PCCl3 thyroid cells by using MitoSOX Red [41]. Mitochondria are potent producers of superoxide, from complexes I and III of the electron transport chain. Mitochondrial superoxide production is a major cause of cellular oxidative damage [42]. Physiologically, ROS are not necessarily harmful because they are continuously balanced by the process of hormone synthesis and the endogenous antioxidant system [1]. Excess ROS are generated during the trapping, oxidation, and organification of excessive iodine in thyrocytes, which could lead to increased oxidative stress [1].

The Nrf2-Keap 1 pathway is the chief cytoprotective mechanism in response to oxidative stress caused by ROS. During normal and balanced redox homeostasis, the Nrf2 function is inhibited because of constant proteasomal degradation after ubiquitination of the protein. This is regulated through the binding of the inhibitor protein Keap 1 [13, 14]. It is reported that Srx activation via Nrf2 dependent pathway protects from oxidative liver injury through Pyrazole [43] and alcohol in mice [44]. Similar findings in lung tissues have shown that there is a marked increase in the expressions of Srx and Prx 3 in human squamous cell carcinoma [45]. This suggests that these proteins may play a protective role against oxidative injury. Also, the pathway including Keap 1, Nrf2, and ARE-mediated protein expression plays a very critical role in protecting cells from oxidative stress [46, 47]. Focusing on the pathway, we demonstrated that the expression of Nrf2 was significantly increased, while Keap 1 was significantly decreased in the 100 HI group when compared to the NI or 10 HI group on Days 7, 14, and 28. Similarly, Ajiboye et al. showed that rats treated with Chalcone dimers not only increased the expression of Nrf2, but also suppressed cytoplasmic Keap 1 expression [48]. Yang et al. also showed that the downregulation of Keap 1 level may be responsible for the overactivation of Nrf2 [49]. Pang et al. showed that caffeic acid prevents acetaminophen-induced liver injury by activating the Nrf2-Keap 1 antioxidative defense system [50].

In order to verify whether high levels of expression of Nrf2 and its nuclear translocation can upregulate some antioxidant enzymes in the thyroid gland, the expressions of Prx 3 and Srx following the intake of NI, 10 HI, and 100 HI on Days 7, 14, and 28 were measured. We demonstrated that the expressions of Srx and Prx 3 in the 100 HI group were significantly increased when compared to the NI group or 10 HI group on Days 7, 14, and 28. The possible explanations are described as below. Firstly, Srx is a cytosolic protein that is able to translocate to sites where hyperoxidized (inactivated) Prx 3 is located. Therefore, it engages itself in the reactivation of Prx 3 under oxidative conditions [51]. Secondly, Prx 3 is a typical 2-Cys Peroxiredoxin located exclusively in the mitochondrial matrix; it is the principal peroxidase responsible for protecting cells from oxidative damage by reducing peroxides such as H_2_O_2_ [52]. Finally, mitochondria contain 30 times more Prx 3 than glutathione peroxidase; Prx 3 can be classified as an important regulator of mitochondrial H_2_O_2_ [22]. The elevated expression of Prx 3 is associated with the blockage of apoptosis, increasing cell proliferation, and is related to adaptive responses, which are all required to maintain mitochondrial function [53, 54]. Bae et al. have suggested that Prx 3 and Srx jointly protect mice from Pyrazole-induced oxidative liver injury in a Nrf2-dependent manner [43].

The novelty we demonstrated in the present study is that iodide excess induced both oxidative stress and antioxidative defense increases through Nrf2-Keap 1 pathway in the thyroid gland from rats. We extended our established mechanisms by applying the Nrf2-Keap 1 pathway to set up a bridge between oxidative stress and antioxidative defense induced by iodide excess in the thyroid gland. In our previous study, we have established that oxidative stress induced by acute high concentrations of iodide in FRTL cells significantly increases mitochondrial superoxide production [55]. The inhibitors of the mitochondrial respiratory chain complexes I and III are involved in mitochondrial superoxide production. We demonstrated that exposure to 100 *μ*M KI for 2 hours significantly increased mitochondrial superoxide production, enhanced by either 0.5 *μ*M Rotenone (an inhibitor of mitochondrial complex I) or 10 *μ*M Antimycin A (an inhibitor of complex III) [56]. We illustrated that 300 *μ*M PTU (an inhibitor of TPO) attenuated the excessive iodide-induced mitochondrial superoxide production. We showed that 30 *μ*M KClO_4_ (a competitive inhibitor of iodide transport) relieved the production the mitochondrial superoxide induced by iodide excess. We displayed that 10 mU/mL TSH can inhibit excessive iodide-induced strong mitochondrial superoxide production [55]. MT-I and MT-II are mainly involved in the protection of tissue against oxidative stresses; we indicated that metallothionein-I/II knockout mice aggravated mitochondrial superoxide production in thyroid after excessive iodide exposure [24]. In addition, we demonstrated that both the oxidative stress and the antioxidative defense increased simultaneously after high dosages of iodide intake. We suggested that the Nrf2-Keap 1 pathway is vital for the balance between oxidative stress and antioxidative defense induced by iodide excess in the thyroid gland.

Excessive iodide stimulated the Nrf2-Keap 1 pathway and enhanced the antioxidative defense. We found that the urinary iodine concentration of the 100 HI group was about 60–80 times that of the NI group on Days 7, 14, and 28; however the thyroid functions were normal. We proposed that the excessive iodide accumulated in the body may trigger the Nrf2-Keap 1 pathway to maintain a normal thyroid function. We demonstrated that the Nrf2 moves from the cytoplasm to the nucleus under the microscope, with significantly increased expressions of Nrf2, Srx, and Prx 3 and notably decreased Keap 1 when exposed to high iodide. This suggests that excessive iodide stimulates the disassociation of Nrf2 from Keap 1 and assists Nrf2 to penetrate the nucleus. Then, Nrf2 attaches to the antioxidant response element (ARE) to activate the expression of the antioxidative genes, Srx and Prx 3, resulting in an enhanced antioxidative defense induced by high iodide. By activation of the Nrf2-Keap 1 pathway, there is a proportional increase in oxidative stress and antioxidative defense in response to iodide excess. It is to be noted that the thyroid function was normal in the 10 HI group and 100 HI in present study. Inspired by the report by Poncin et al. [6], we proposed that there should be a balance between oxidative stress and antioxidative defense in response to iodide excess in the 10 HI and the 100 HI groups.

## 5. Conclusion

**Figure 5:**
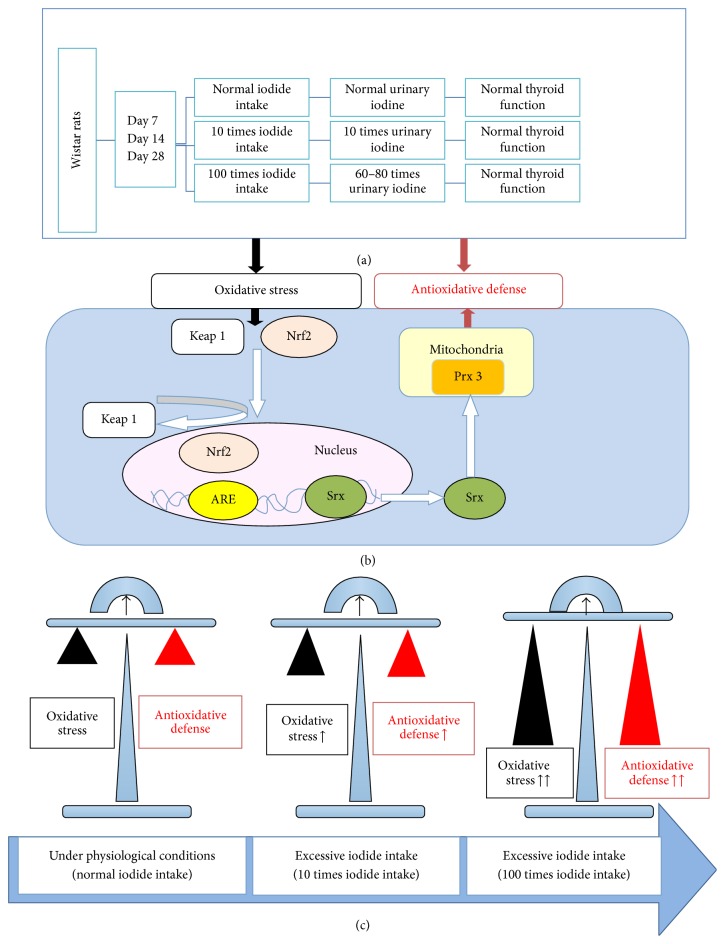
Proposed mechanisms in the present study. (a) The urinary iodine concentration and thyroid function of Wistar rats were detected. (b) The activation of the Nrf2-Keap 1 pathway induced by iodide excess in the thyroid. (c) The balance between oxidative stress and antioxidative defense under physiological conditions and excessive iodide intake.

In conclusion, our results highlight that the activation of Nrf2-Keap 1, Srx, and Prx 3 antioxidative defense mechanisms may play a crucial role in protecting the thyroid from iodide excess induced oxidative stress on Days 7, 14, and 28 (Figure 5).

## Figures and Tables

**Table 1:** Changes of the median urinary iodine concentration (*μ*g/L) of Wistar rats following the intake of NI, 10 HI, and 100 HI on Days 7, 14, and 28 (*N* = 6 for each group). All data is presented as the median (range).

Group	The median urinary iodine concentration (*μ*g/L)		
	Day 7	Day 14	Day 28
NI	321.2 (150.0–351.7)	358.2 (300.2–373.8)	333.0 (188.6–413.5)
10 HI	3052.5^**∗**^ (1592.8–3798.0)	3532.5^**∗**^ (1487.0–4056.0)	2628.0^**∗**^ (1651.0–3404.5)
100 HI	26489.2^**∗**#^ (5856.6–42170.4)	24461.0^**∗**#^ (17607.5–29874.8)	22663.2^**∗**#^ (8342.5–29816.2)

^**∗**^Compared to the NI group (*p* < 0.05).

^#^Compared to the 10 HI group (*p* < 0.05).

**Table 2:** Changes of iodine content in the thyroid glands (*μ*g/100 mg) of Wistar rats following the intake of NI, 10 HI, and 100 HI on Days 7, 14, and 28 (*N* = 6 for each group). All data is presented as the mean ± SD.

Group	Iodine content in the thyroid glands (*μ*g/100 mg)		
	Day 7	Day 14	Day 28
NI	62.19 ± 5.94	60.12 ± 8.13	63.37 ± 9.08
10 HI	121.25 ± 10.48	126.45 ± 8.93	152.33 ± 10.77
100 HI	133.53 ± 8.61^**∗**^	136.36 ± 9.64^**∗**^	178.45 ± 8.74^**∗**^

^**∗**^Compared with NI group (*p* < 0.05).

**Table 3:** Changes of serum thyroid hormones levels of Wistar rats following the intake of NI, 10 HI, and 100 HI on Days 7, 14, and 28 (*N* = 6 for each group). All data is presented as the mean ± SD.

Group	The levels of serum thyroid hormones			
	TT3 (nmol/L)	TT4 (nmol/L)	FT3 (pmol/L)	FT4 (pmol/L)
Day 7				
NI	1.18 ± 0.15	90.77 ± 5.52	3.96 ± 0.10	22.03 ± 1.70
10 HI	1.19 ± 0.08	91.54 ± 7.74	3.77 ± 0.26	23.02 ± 2.06
100 HI	1.07 ± 0.11	89.17 ± 9.03	3.78 ± 0.11	20.08 ± 5.43
Day 14				
NI	1.15 ± 0.07	95.83 ± 5.20	3.92 ± 0.16	22.34 ± 3.71
10 HI	1.12 ± 0.08	86.15 ± 2.52	3.63 ± 0.48	20.19 ± 1.92
100 HI	1.19 ± 0.04	91.54 ± 3.31	3.84 ± 0.46	20.98 ± 2.50
Day 28				
NI	1.13 ± 0.06	94.17 ± 2.38	3.89 ± 0.13	20.25 ± 5.72
10 HI	1.18 ± 0.08	90.77 ± 3.78	3.98 ± 0.57	21.15 ± 5.34
100 HI	1.14 ± 0.22	87.69 ± 6.35	3.79 ± 0.46	21.17 ± 5.43

## Abbreviations

NI: Normal iodide intake
HI: High iodide intake
ROS: Reactive oxygen species
H_2_O_2_: Hydrogen peroxide
Nrf2: Nuclear factor erythroid 2-related factor 2
Keap 1: Kelch-like ECH-associated protein 1
ARE: Antioxidant response element
Prx: Peroxiredoxin
Srx: Sulfiredoxin
Prx 3: Peroxiredoxin 3
TT4: Total thyroxine
TT3: Total triiodothyronine
FT4: Free thyroxine
FT3: Free triiodothyronine
TSH: Thyrotropin
LSD: Least significant difference.
